# Supplementary material for: Endothelial TRIM47 regulates blood-brain barrier integrity and cognition via the KEAP1/NRF2 signalling pathway in mice
Source: Commun Biol. 2026 Feb 10;9:399. doi: 10.1038/s42003-026-09628-5 (PMC13000003; doi:10.1038/s42003-026-09628-5)
Supplement: Supplementary file 2 — Supplementary information [file 42003_2026_9628_MOESM2_ESM.pdf]

Supplementary Information

Endothelial TRIM47 regulates blood-brain barrier integrity and cognition *via* the KEAP1/NRF2 signalling pathway in mice

Authors: Valentin Delobel[1,$], Camille Grenier[1,$], Romain Boulestreau[1], Sébastien Rubin[1], Juliette Vaurs[1], Béatrice Jaspard-Vinassa[1], Elina Casas[1], Muriel Busson[1], Cloé Combrouze[1], Carole Proust[2], Ilana Caro[2], Jean-Luc Morel[3], Bruno Bontempi[3], Aniket Mishra[2], Stéphanie Debette[2], Cécile Duplàa[1], Thierry Couffinhal[1, #] and Claire Peghaire[1, #,*].

[1] Univ. Bordeaux, INSERM, Biologie des maladies cardiovasculaires, U1034, F-33600 Pessac, France
[2] Univ. Bordeaux, INSERM, Bordeaux Population Health, U1219, F-33000 Bordeaux, France
[3] CNRS, INCIA, UMR5287, University Bordeaux, F-33000, Bordeaux, France

[$] These authors contributed equally
[#] These authors jointly supervised this work
[*] email of corresponding author: claire.peghaire@u-bordeaux.fr

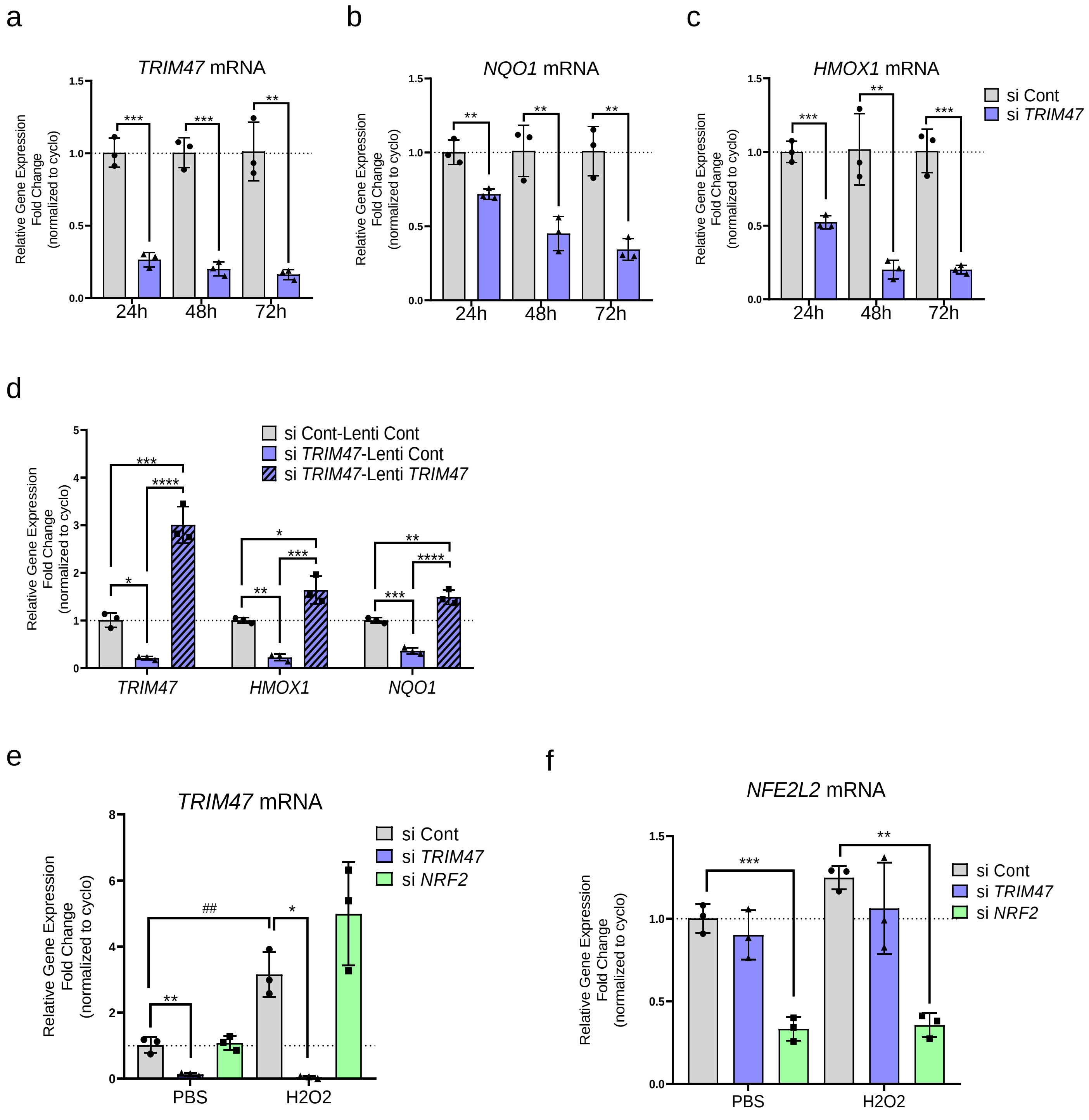

Supplementary Figure 1: Regulation of NRF2 target genes by TRIM47 in human brain microvascular endothelial cells (HBMEC). **a-c**. qPCR analysis of (**a**) *TRIM47*, (**b**) *NQO1* and (**c**) *HMOX1* expression in control (siCont) and *TRIM47*-deficient (si*TRIM47*) HBMEC after 24, 48 and 72 hours siRNA treatment. Data were normalized to cyclophilin (n=3 experiments). **d**. qPCR analysis of *TRIM47*, *HMOX1 and NQO1* expression in control or *TRIM47* siRNA-treated HBMEC (48 h) and co-transduced a control (Lenti Cont) or TRIM47 lentivirus (Lenti T*RIM47*) for 24h. Data were normalized to cyclophilin (n=3 experiments). * P<0.05; ** P<0.01; *** P<0.001, **** P<0.0001, One-way ANOVA. **e-f**. qPCR analysis of (**e**) *TRIM47* and (**f**) *NFE2L2* (NRF2) gene expression in control, *TRIM47* or *NRF2* siRNA-treated HBMEC for 72h and treated with either PBS or H2O2 (200 uM for 3h). Data were normalized to *cyclophilin* (n=3 experiments). * P<0.05; ** P<0.01; *** P<0.001, One-way ANOVA, ## P<0.01, Student's *t*-test. All Graphical data are mean ± s.d.

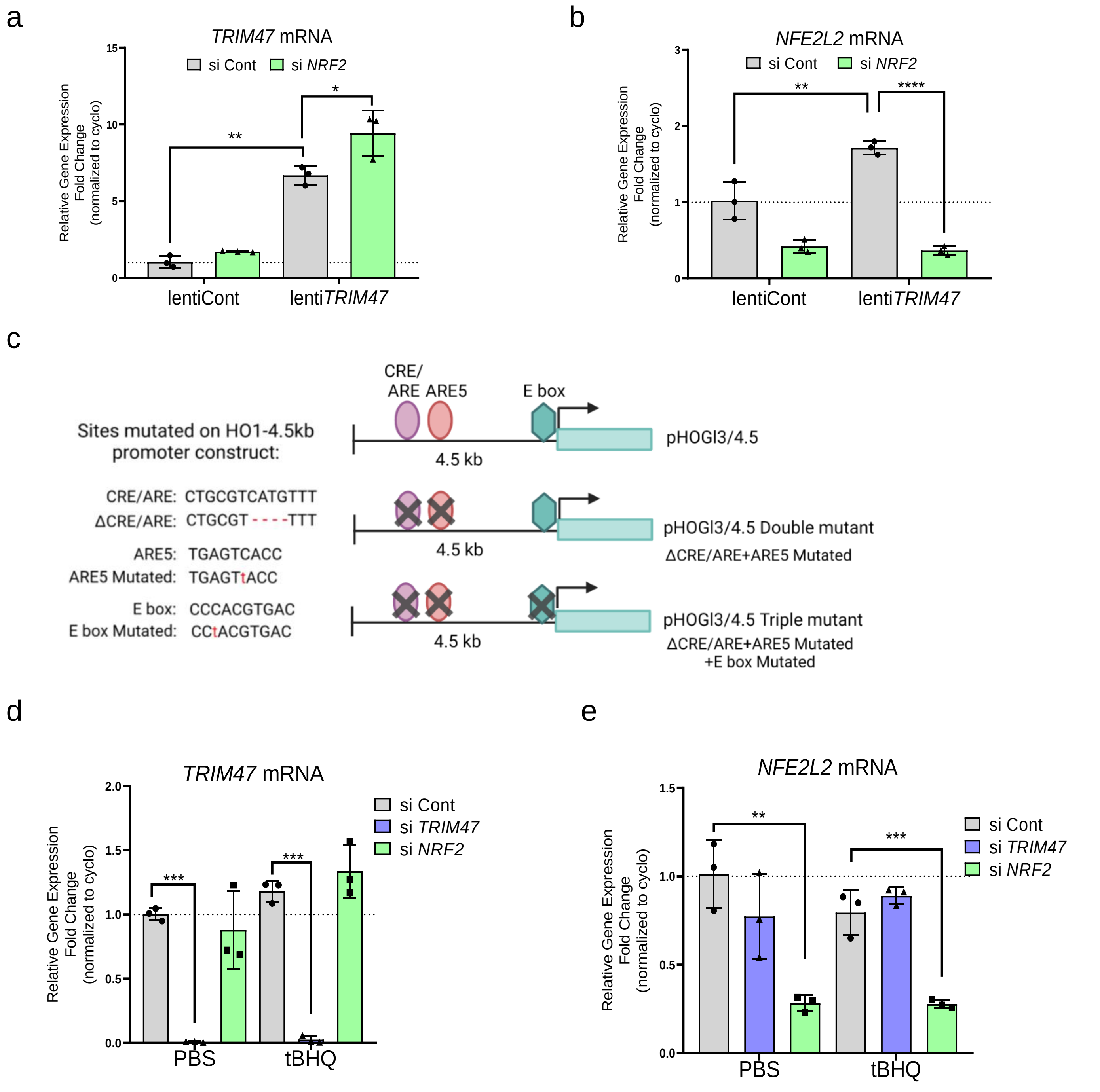

Supplementary Figure 2: Controls and tools for the experiments showing TRIM47 cooperation with the transcription factor NRF2 in HBMEC. **a-b**. qPCR analysis of (**a**) *TRIM47* and (**b**) *NFE2L2* expression in siCont or si*NRF2* treated HBMEC for 48h and co-transduced with a control or TRIM47 lentivirus for 24h. Data were normalized to *cyclophilin* (n=3 experiments). ** P<0.01; *** P<0.001; One-way ANOVA. **c.** Cartoon depicted the HO1 tools used for promoter luciferase reporter assay in HeLa. Wild type HO1 promoter-luciferase construct (HO1-4.5bp), construct mutated for CRE/ARE and ARE5 sites (HO1-4.5bp double mutant) and construct mutated for CRE/ARE, ARE5 sites and E box (HO1-4.5bp triple mutant, used as a control for the experiment). Created in BioRender. Duplaa, C. (2026) huffp8m https://BioRender.com/. **d-e**. qPCR analysis of (**d**) *TRIM47* and (**e**) *NFE2L2* expression in HBMEC treated with control, *TRIM47* or *NRF2* siRNA for 72h and with tBHQ for 24h (n=3 experiments). ** P<0.01; *** P<0.001; One-way ANOVA. All Graphical data are mean ± s.d.

a

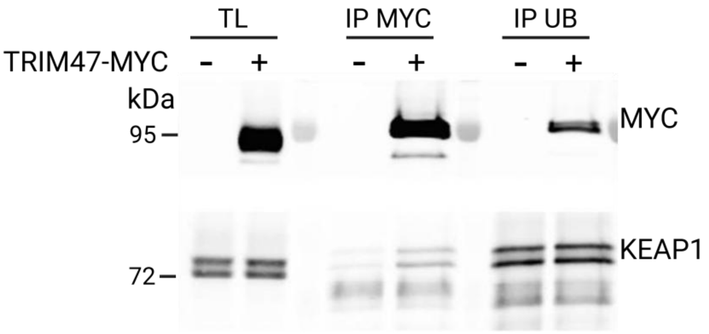

b

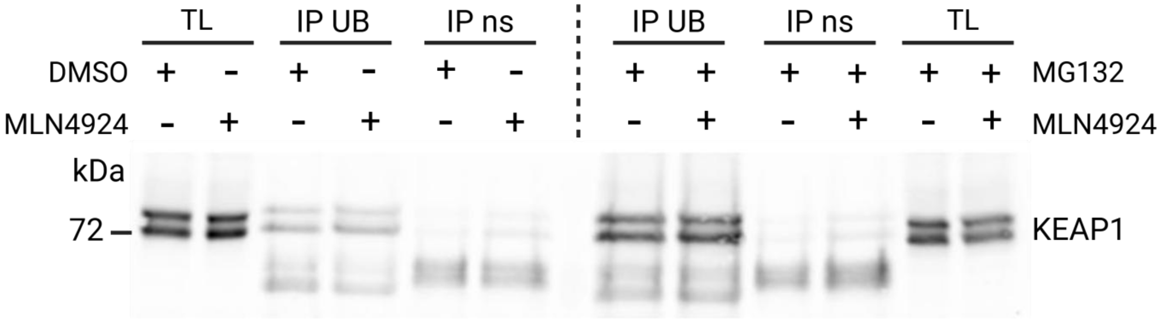

Supplementary Figure 3: TRIM47 does not induce KEAP1 ubiquitination. **a.** TRIM47 and KEAP1 interaction, as well as, KEAP1 ubiquitination were assessed by semi-endogenous Co-IP assay in whole cell lysates from HEK293. Cells were transfected with TRIM47-myc pcDNA. Lysates were immunoprecipitated with myc antibody or Ub antibody and immuno-blotted for myc (TRIM47) and KEAP1. **b.** Contribution of the cullin-RING ligases on KEAP1 ubiquitination was assessed by Co-IP assay in HEK293 cells treated with MLN4924 (selective NEDD8-activated enzyme inhibitor, 0.1µM, for 4h) and co-treated with either DMSO or MG132 (10µM). Lysates were immunoprecipitated with Ub antibody and immuno-blotted for and KEAP1. TL: total lysates; IP ns: non specific.

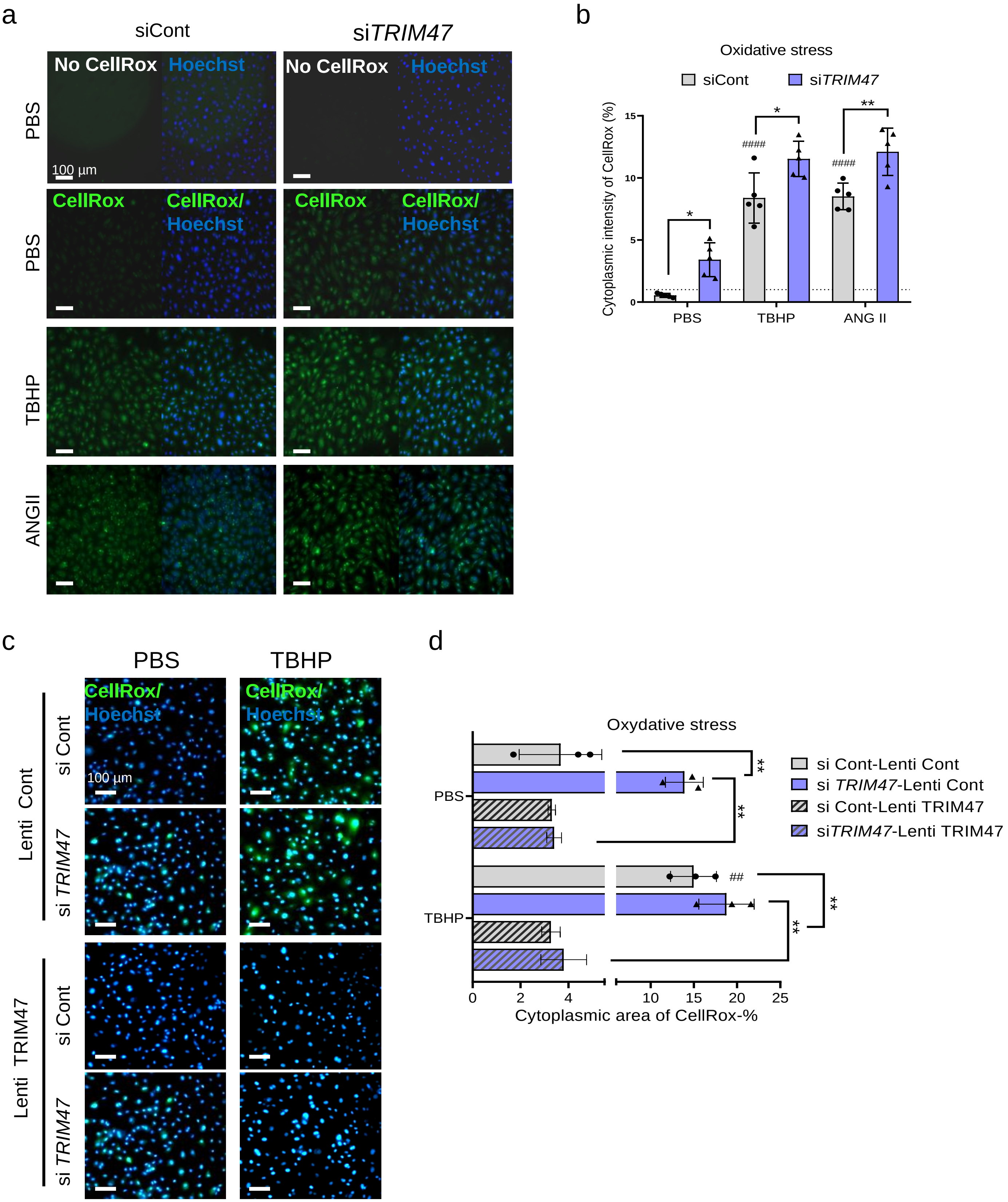

Supplementary Figure 4: TRIM47 displays antioxidant properties in HBMEC. **a-b.** Representative immunofluorescence image (**a**) and quantification (**b**) of CellRox dye (green) in HBMEC transfected with siCont or si*TRIM47* for 24 h and treated with PBS or tert-butyl hydroperoxide (TBHP, 200 µM for 1 h) or Angiotensin II (ANGII, 500 nM for 2h). Quantification represents the mean percentage of cytoplasmic area of Cellrox dye per field (n=3-5 wells from 3 independent experiments). **c-d.** Representative immunofluorescence image (**c**) and quantification (**d**) of CellRox dye (green) in HBMEC transfected with siCont or si*TRIM47* for 48 h, co-transduced with control or TRIM47 lentivirus for 24h and treated with PBS or tert-butyl hydroperoxide (TBHP, 200 µM for 1 h). Quantification represents the mean percentage of cytoplasmic area of Cellrox dye per field (n=3 independent experiments). Nuclei are identified by Hoechst (blue). Scale bar 100 µm. * P<0.05, ** P<0.01, One-way ANOVA; ## P<0.01: #### P<0.0001: compared to siCont + THBP without drug treatment. Graphical data are mean ± s.d.

a

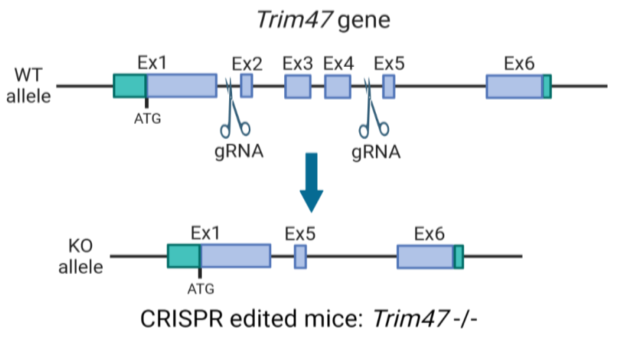

b

genotype	+/+	-/-	Total
% observed	23	22	100
% -mendelian ratio	25	25	100

c d e

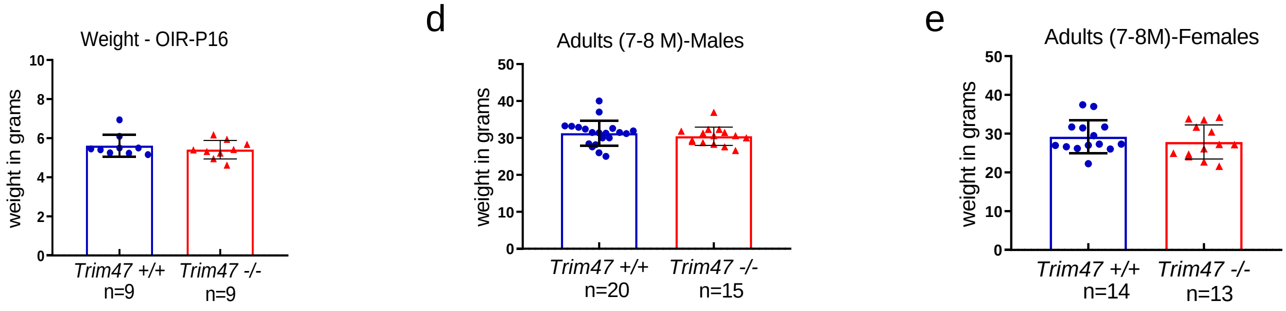

f

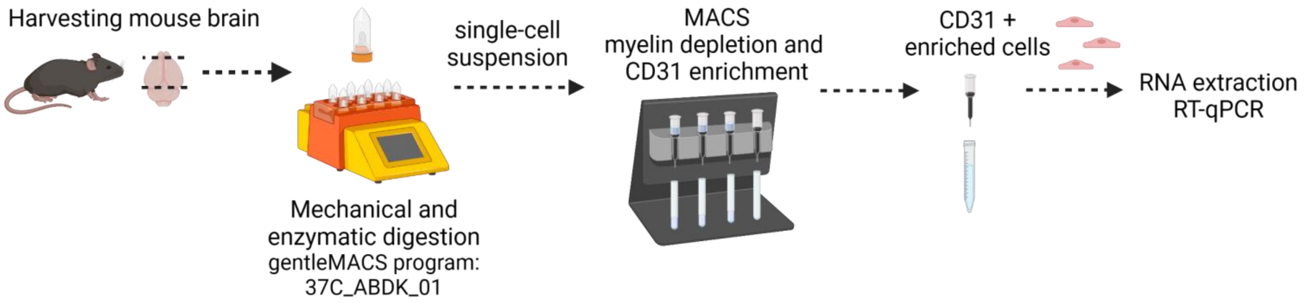

g

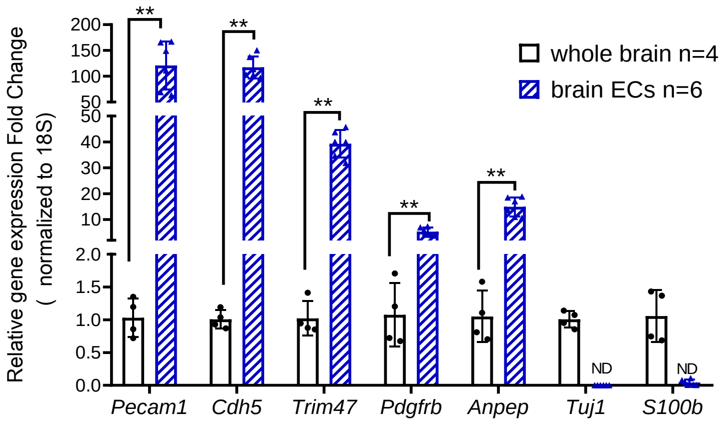

Supplementary Figure 5: Generation and characterization of mice deleted for *Trim47* in all tissues. **a.** Cartoon depicting the strategy for generating mice globally deleted for *Trim47* using CRISPR deletion of exons 2 to 4. Created in BioRender. Duplaa, C. (2026) https://BioRender.com/m35wbbr. **b**. Expected Mendelian ratio and percentage of mice observed with +/+ or -/- genotype is reported in the table. **c-e.** Body weight (in grams) of (**c**) postnatal day 16 mice after OIR model, adult (**d**) males and (**e**) females +/+ and -/- mice. **f.** Workflow of mouse brain endothelial cells isolation protocol using gentle MACS for mechanical and enzymatic digestion, then myelin removal step and enrichment with CD31 magnetic beads. Created in BioRender. Duplaa, C. (2026) https://BioRender.com/ylfh2mh. **g.** qPCR analysis of whole brain lysates and brain endothelial cells (ECs) for endothelial (*Pecam1*, *Cdh5*, *Trim47*) , pericytes (*Pdgfrb*, *Anpep*), neurons (*Tuj1*) and myelin (*S100b*) markers to show purity of isolated ECs. n=4 mice (7-10 months) for whole brain samples; n=6 samples for brain ECs (2 mice pooled per sample, 7-10 months). Data normalized to *18S.* ND: not detectable; * * P<0.01, Mann-Whitney. All gaphical data are mean ± s.d.

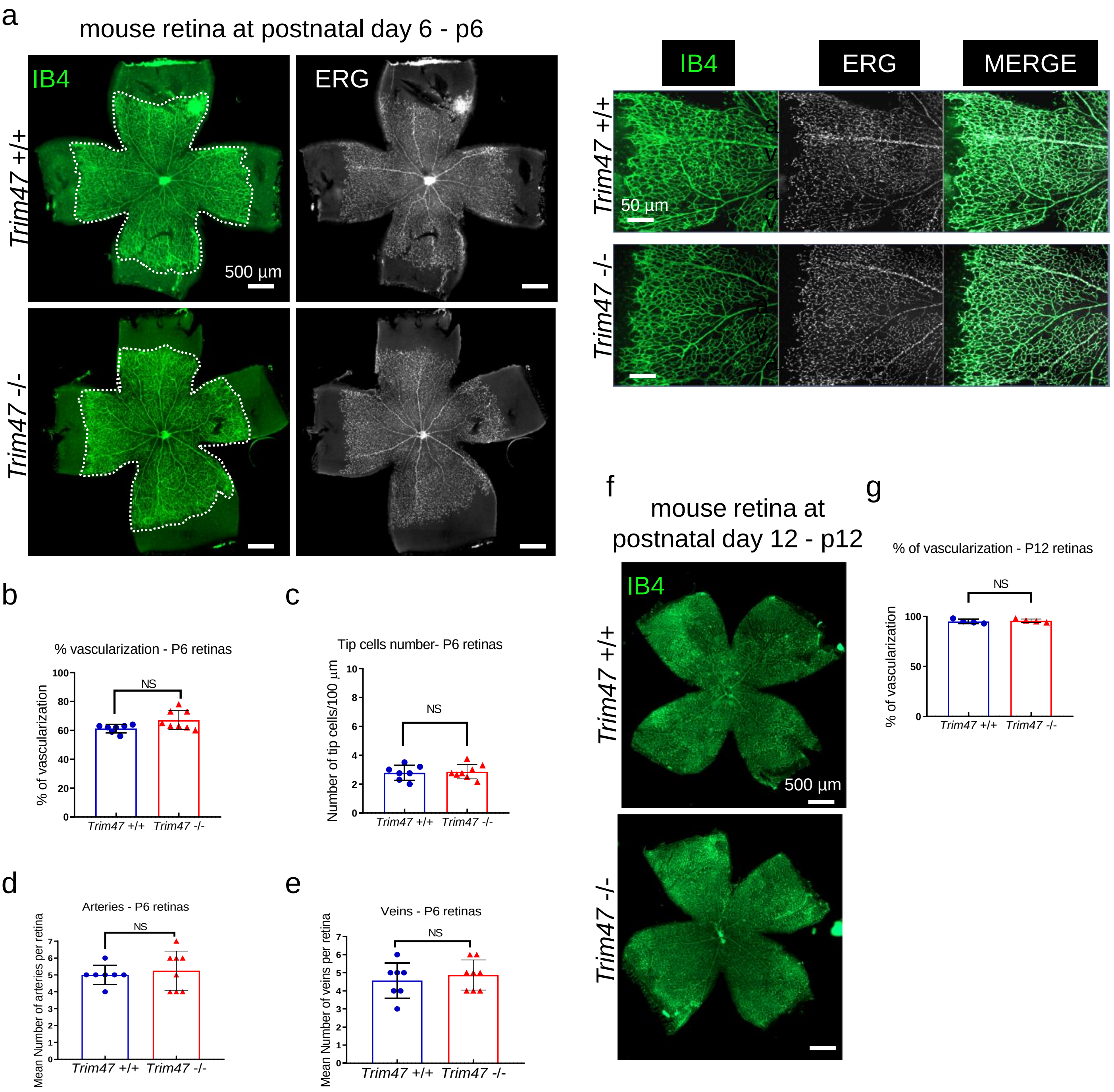

Supplementary Figure 6 : *Trim47* deletion has no impact on postnatal angiogenesis in mouse retina. **a.** Representative images of isolectin B4 (IB4, green; blood vessels) and ERG (white; endothelial cells) staining of postnatal day 6 retinas from *Trim47*+/+ and *Trim47*-/- mice. Left panels show widefield images of whole retinas, and right panels show higher magnification views. Dashed white lines delineate the vascularized area. Scale bars: 500 µm and 50 µm. **b-e**. Quantification represents (**b**) the percentage of vascularized area, (**c**) the number of tip cells and the number of (**d**) arteries and (**e**) veins in *Trim47*+/+ (n=7) and *Trim47*-/- mice (n=8) at p6. **f.** Representative images of isolectin B4 (IB4, green; blood vessels) staining of postnatal day 12 retinas from *Trim47*+/+ and *Trim47*-/- mice. Scale bars: 500 µm. **g .** Quantification represents the percentage of vascularized area in p12 retinas from *Trim47*+/+ (n=4) and *Trim47*-/- (n=4) mice.. a: artery; v: vein. Mann Whitney test. NS: not significant, p>0.05. All graphical data are mean ± s.d.

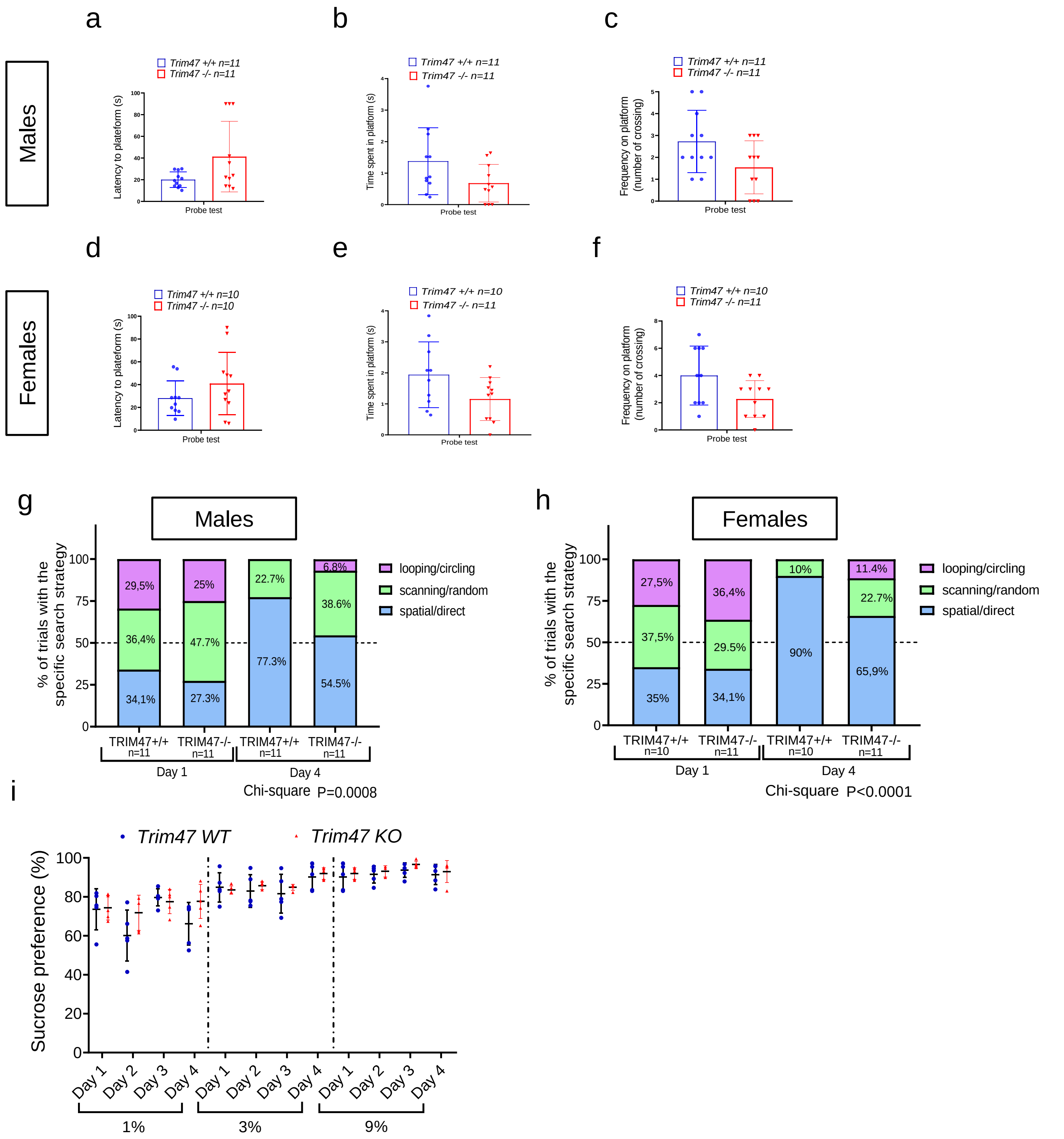

Supplementary Figure 7: Assessment of cognitive functions and anhedonia of mice deleted for *Trim47.* **a-f.** Probes trials were performed 3 days after the last training on males (**a-c**) and females (**d-f**). The 3 additional following parameters were measured: (**a-d**) latency to reach platform area (seconds), (**b-e**) time spent in platform area (seconds) and (**c-f**) frequency on platform (number of platform crossings). In line with an increase in proximity measure, a trend towards increased latency to reach platform area, decreased time spent in platform area and number of crossings were observed in *Trim47-/-* mice. *Trim47+/+* males: n=11, *Trim47-/-* males: n=11.males, *Trim47+/+* females: n=10, *Trim47-/-* females: n=11. * P<0.05, Mann-Whitney test. **g-h.** Search strategy use in water maze was affected in *Trim47-/-* mice. Males (**g**) and females (**h**) were analyzed separately and classified in 3 categories: spatial/direct, scanning/random and looping/circling. Data from all trials were combined for this analysis and expressed as percentage of trials using each strategy for each genotype at days 1 and 4 of training. *Trim47+/+* n=44 trials (11 males)*; Trim47-/-* n=44 trials (n=11 males) (4 trials/mouse). *Trim47+/+* n=40 trials (10 females)*; Trim47-/-* n=44 trials (n=14 females). Males: p=0.008 and females: p<0.0001. Chi-square test. **i.** Sucrose preference test was performed to asses anhedonia and anxiety in adults +/+ (blue dots) and -/- (red triangles) mice (males, 8 months, n=5 mice per genotype) and is expressed as the percentage of sucrose intake normalized to the total amount of liquid (water and sucrose) intake. Graphical data are mean ± s.d.

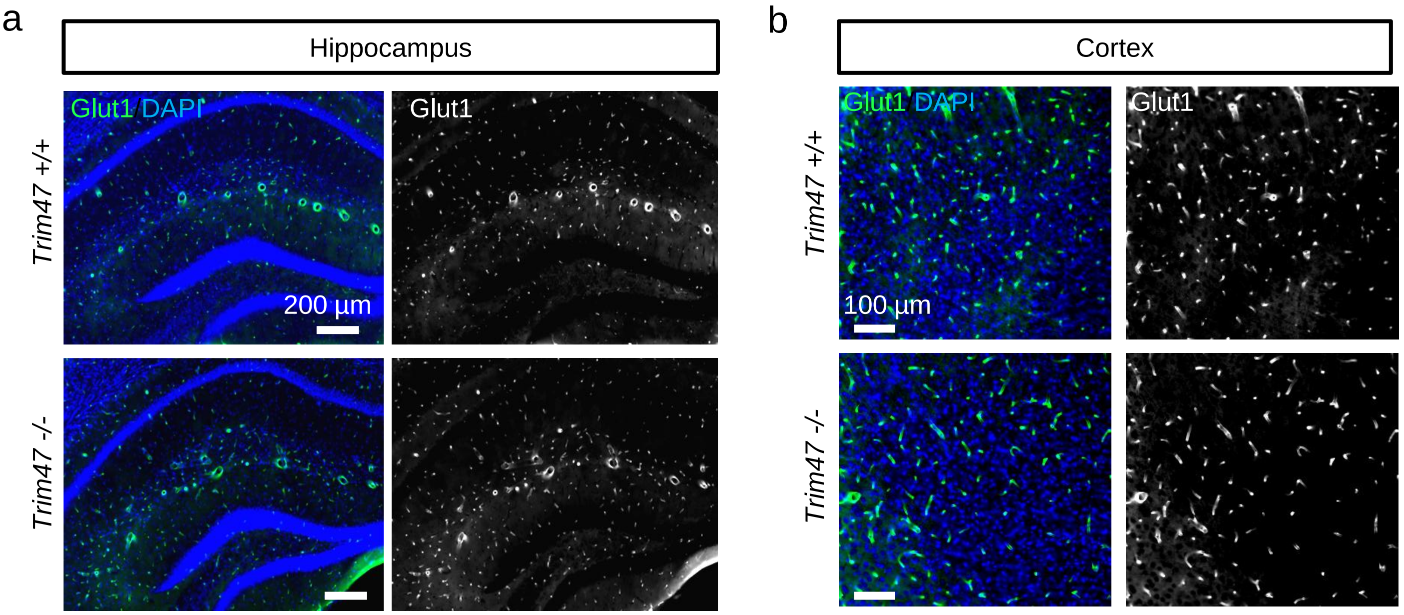

c Permeability - 0,95kDa cadaverin

	raw data serum RFU	raw data brain RFU	x10^-3 ml/g brain PI
WT 1	4691,50	23,75	0,53
WT 2	7732,50	35,75	0,59
WT 3	8145,50	26,75	0,40
WT 4	4797,50	10,75	0,26
WT 5	4995,50	9,75	0,20
KO 1	4842,50	32,75	0,71
KO 2	7318,50	43,75	0,75
KO 3	7901,50	53,75	0,81
KO 4	3995,50	23,75	0,66
KO 5	4519,50	20,75	0,60

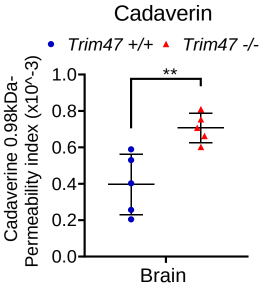

d Permeability - 3kDa dextran

	raw data serum RFU	raw data brain RFU	x10^-2 ml/g brain PI
WT 1	1091,50	133,75	1,46
WT 2	1037,50	132,75	1,42
WT 3	976,50	148,75	1,54
WT 4	981,50	59,75	0,75
WT 5	1026,50	127,75	1,60
KO 1	1202,50	163,75	1,72
KO 2	979,50	135,75	1,83
KO 3	1324,50	254,75	2,29
KO 4	1232,50	256,75	2,57
KO 5	1129,50	139,75	1,81

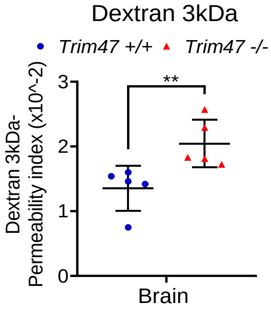

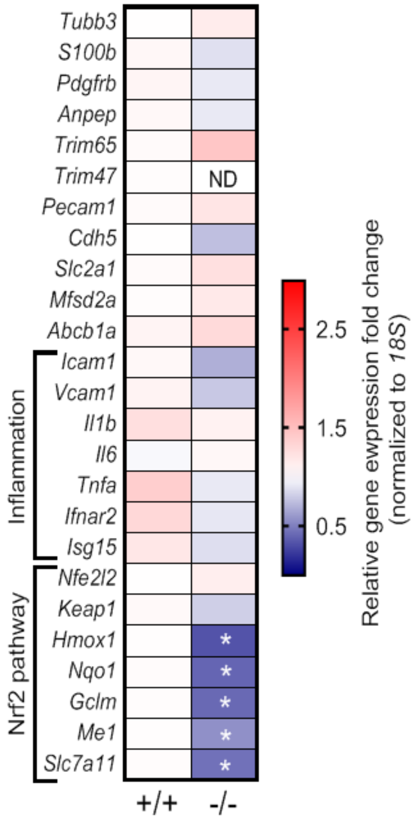

Supplementary Figure 8: Histological and transcriptomic analysis of *Trim47*+/+ and *Trim47-/-* adults mice. **a-b.** Representative images of Glut1 (green or grayscale) in brain sections (coronal, 50 um) from *Trim47*+/+ and *Trim47*-/- mice showing blood vessels in (**a**) hippocampus and (**b**) cortical regions. Nuclei are stained with DAPI (blue). Scale bars 200 or 100 µm, as noted. **c-d.** Tables with raw fluorescence units (RFU) and graphs for non normalized brain permeability index (PI) for cadaverin (**c**) and 3kDa dextran (**d**) tracers in *Trim47*+/+ and *Trim47*-/- brains (n=5 per genotype). ** P<0.01, Mann-Whitney test. **e**. qPCR screening of whole brain from *Trim47*+/+ and *Trim47*-/- mice (7-10 months). Data normalized to *18S* (n=4 *Trim47*+/+, n=4 *Trim47* /-). ND: not detectable; * P<0.05, Mann-Whitney test. All Graphical data are mean ± s.d.

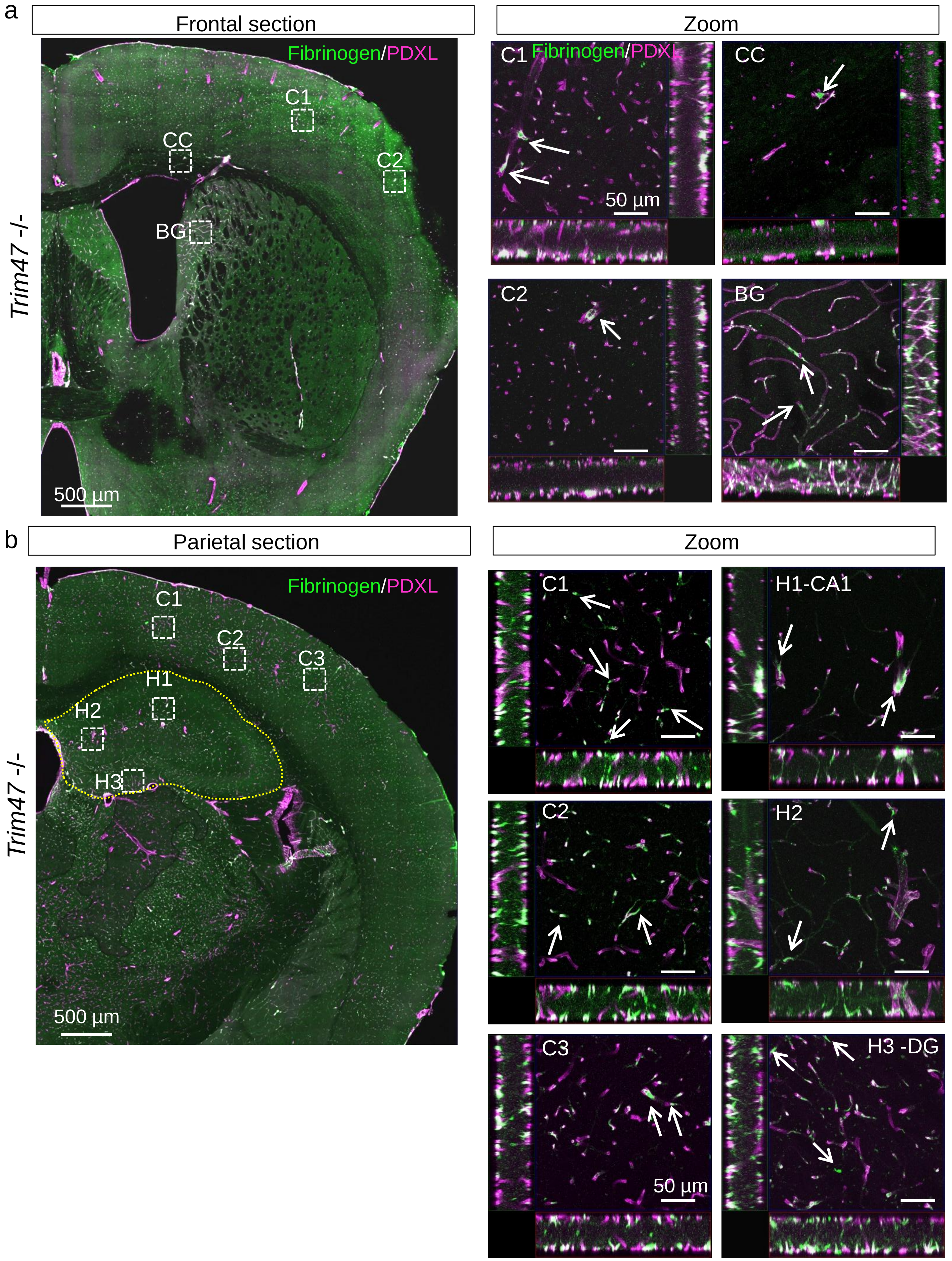

Supplementary Figure 9: Localization of fibrinogen leakages in *Trim47* -/- mice. **a-b** Representative image of fibrinogen staining (green) in (**a**) frontal and (**b**) parietal brain sections (coronal, 50 um) of *Trim47* -/- mice (8 months old). Widefield images and higher-magnification views are presented. Tissues were co-stained with podocalyxin (PDXL; pink) to visualize blood vessels, and white arrows indicate fibrinogen leakage in various brain regions. Dashed yellow line delineates the hippocampus. Scale bars 500 or 50 µm. C: cortex; CC: corpus callosum; BG: basal ganglia; DG: dentate gyrus; H: hippocampus.

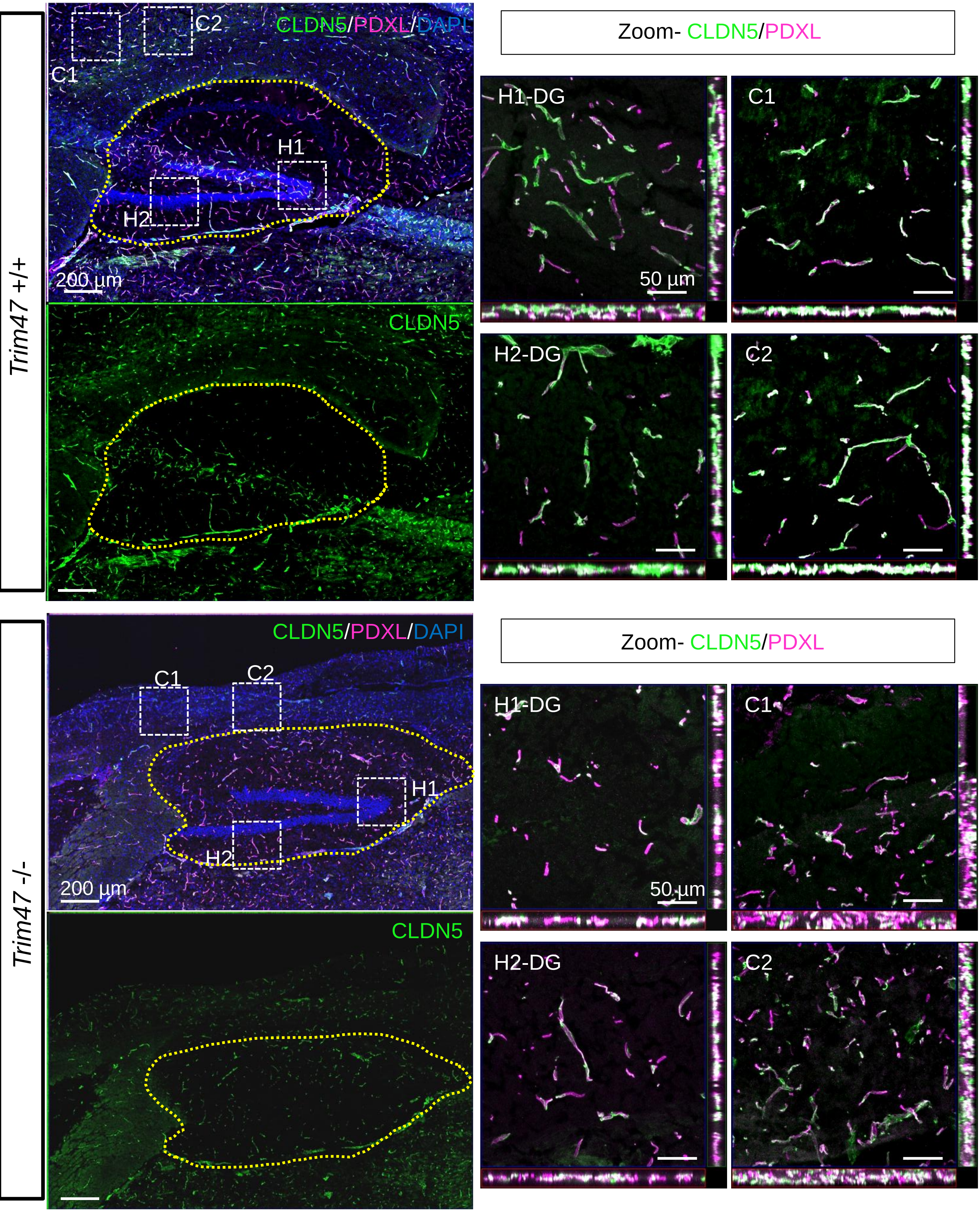

Supplementary Figure 10: Representative image of claudin5 staining (CLDN5: green) in sagittal brain sections (10 um) of *Trim47* +/+ and *Trim47* -/- mice (8 months old). Widefield images and higher-magnification views (zoom) in different brain regions are presented. Tissues were co-stained with podocalyxin (PDXL; pink) to visualize blood vessels; nuclei identified by DAPI (blue). Scale bars: 200 µm or 50 µm. Dashed yellow lines delineate the hippocampus. C: cortex; DG: dentate gyrus; H: hippocampus.

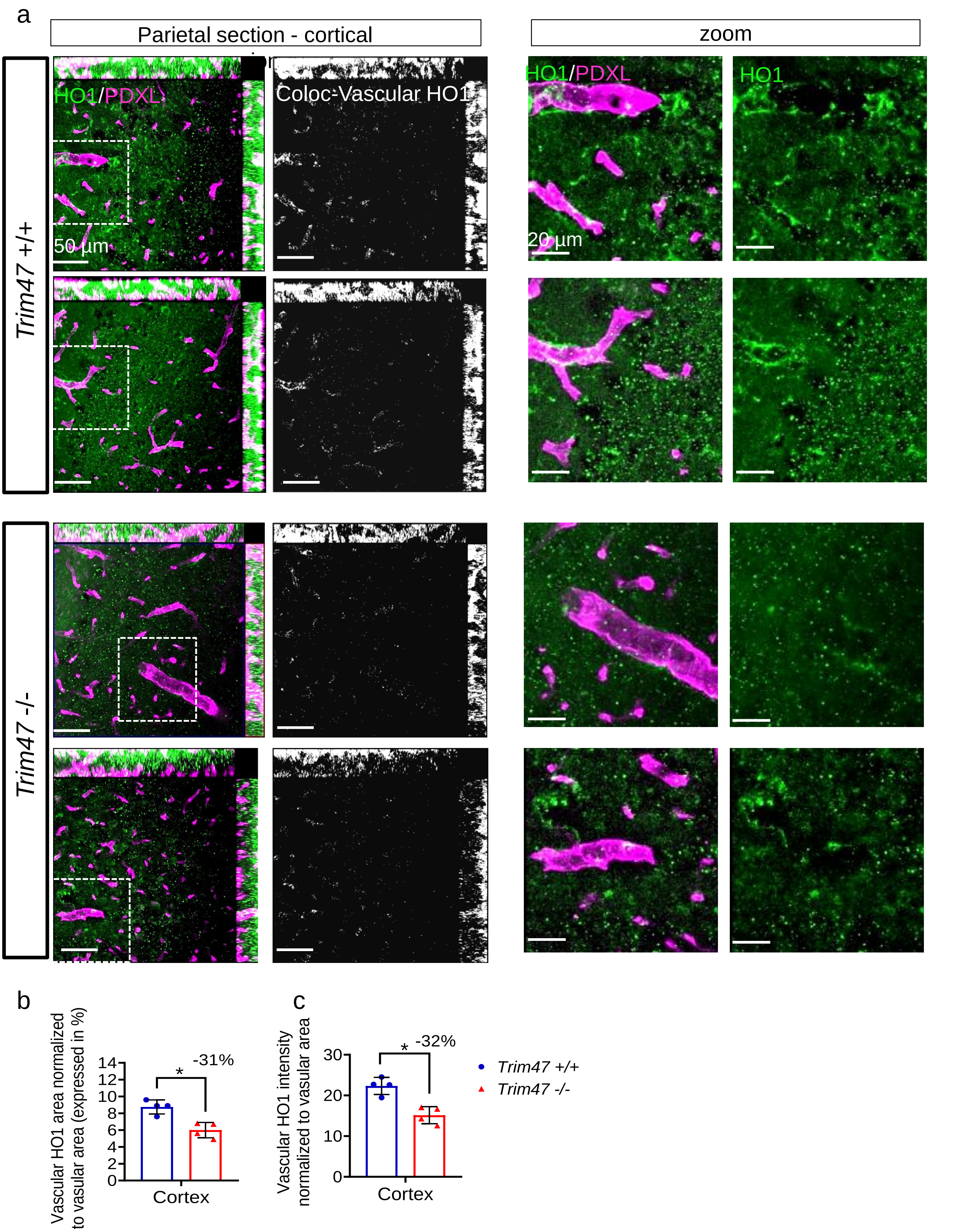

Supplementary Figure 11: **a.** Representative confocal images of HO1 immunostaining (green) in parietal brain sections (coronal, 50 µm) from *Trim47* +/+ and *Trim47*-/- mice (8 months old). Sections were co-stained with podocalyxin (PDXL; pink) to visualize blood vessels. Merged images and colocalization masks highlighting vascular HO1 ($HO1^+/PDXL^+$ signal; white) and higher-magnification views (zoom) of cortical regions. are shown. Scale bars: 50 µm (low magnification) and 20 µm (high magnification). **b–c.** Quantification of (**b**) vascular HO1 ($HO1^+/PDXL^+$) area and (**c**) intensity, both normalized to total vascular area ($PDXL^+$ area) in the cortex. n = 4 *Trim47* +/+ and n = 4 *Trim47*-/- mice. * P<0.05, Mann–Whitney test. All Graphical data are mean ± s.d.

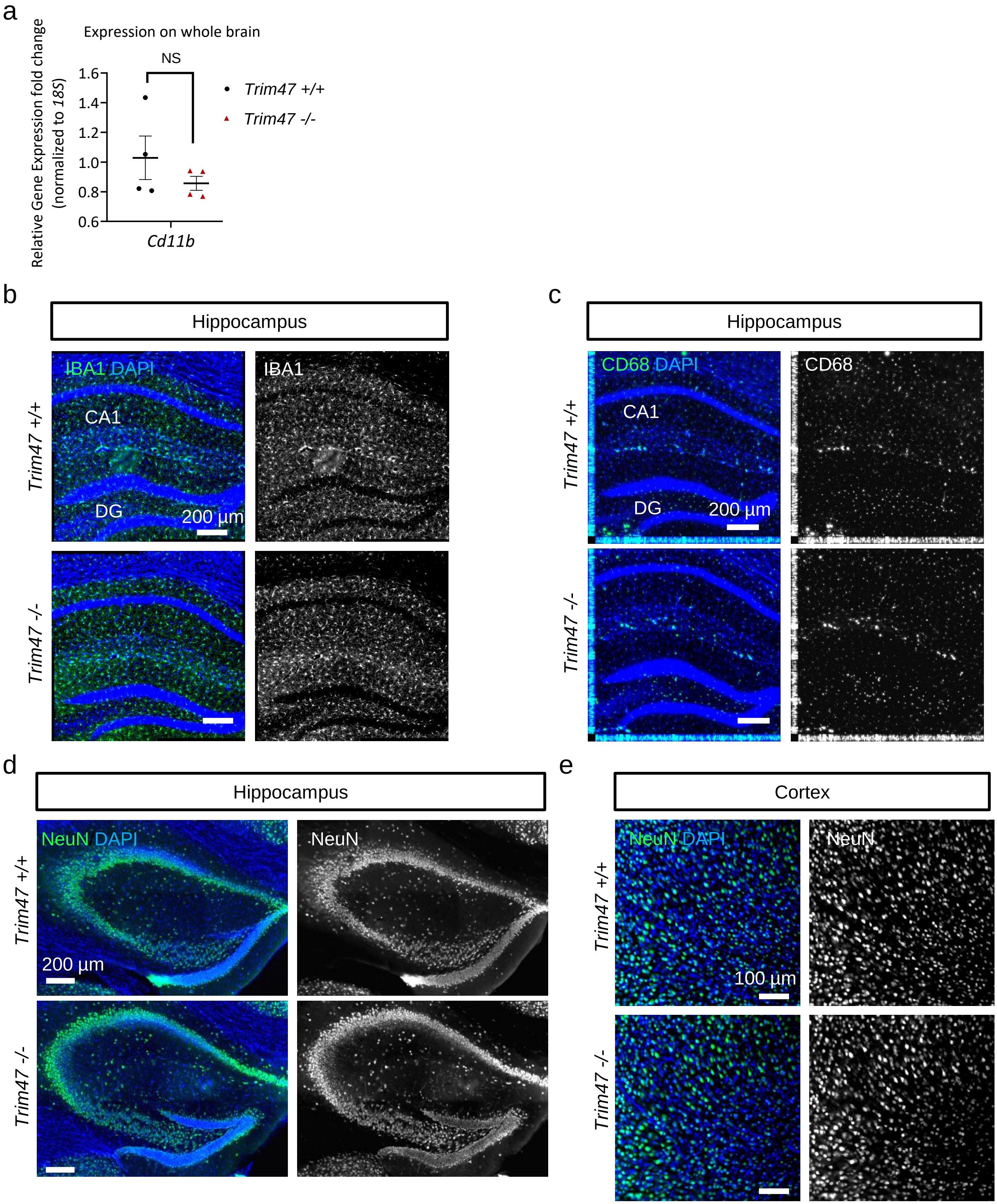

Supplementary Figure 12: Effects of *Trim47* deletion on non-endothelial cells in adult mouse brain.
a. mRNA expression profile of the microglia marker *Cd11b* in whole brain lysates from *Trim47*+/+ and *Trim47*-/- mice (7-10 months). Data normalized to *18S* (n=4 *Trim47*+/+, n=4 *Trim47*-/-). **b-c.** Representative images of (**b**) Iba1 (microglia) (green/grayscale) and (**c**) Cd68 (macrophages) (green/grayscale) immunostaining in brain sections (coronal, 50 um, hippocampus) from *Trim47*+/+ and *Trim47*-/- mice. Scale bars 200 µm. CA1: cornu ammonis region 1, DG: dentate gyrus **d-e**. Representative images of Neun staining (neurons) (green/grayscale) in brain cryosections from *Trim47*+/+ and *Trim47*-/- mice in (**d**) hippocampus and in (**e**) cortex region; nuclei identified by DAPI (blue). Scale bars 200 or 100 µm. Mann Whitney test. NS: not significant, p>0.05. Graphical data are mean ± s.d.

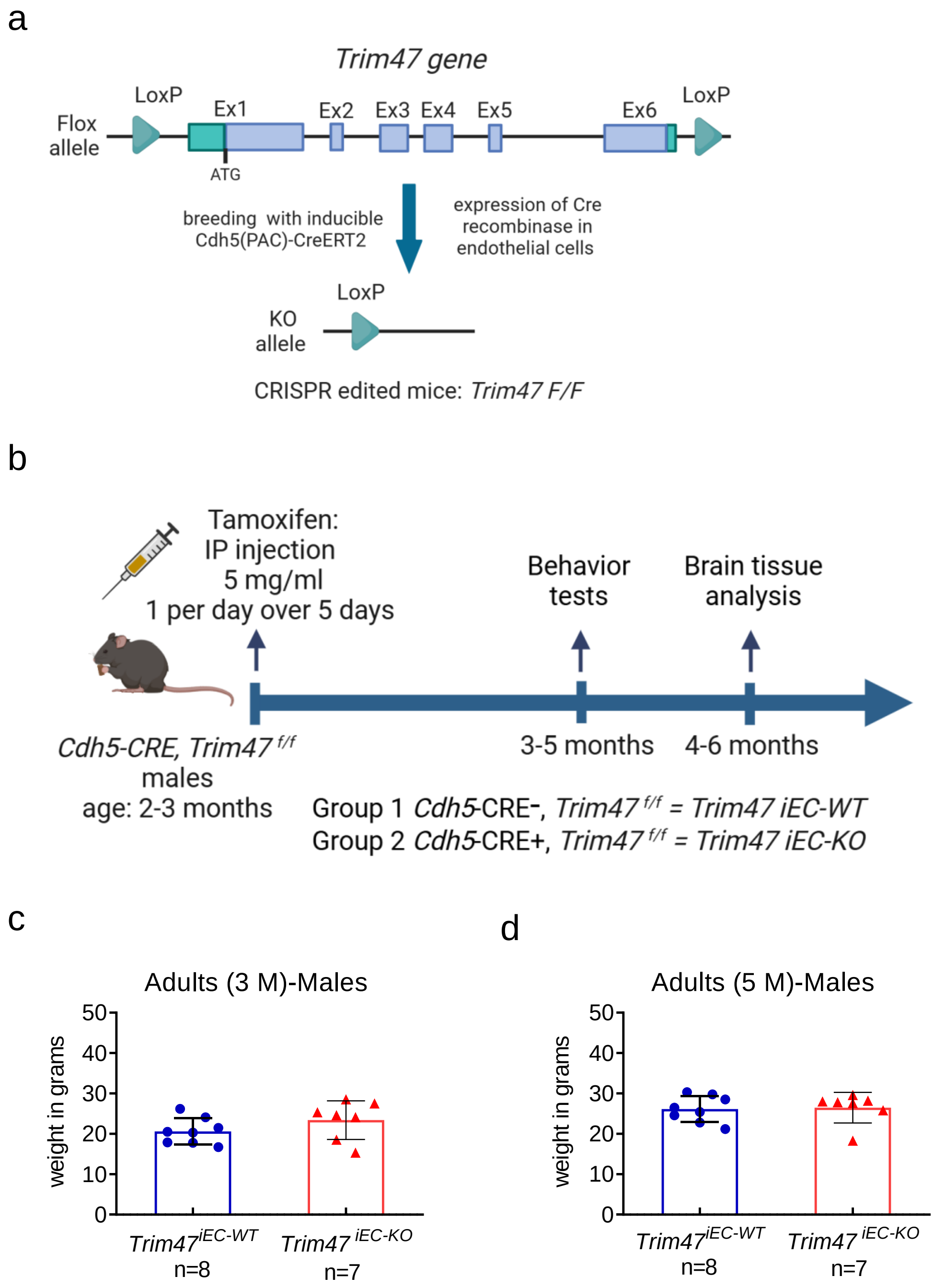

Supplementary Figure 13: Generation and characterization of mice deleted for *Trim47* specifically in endothelial cells. **a.** Cartoon depicting the strategy for generating mice deleted for *Trim47* in endothelial cells using *Trim47 F/F* mice (Flox sequences flanking exons 1 to 6) bred with *Cdh5*-Cre mice. Created in BioRender. Duplaa, C. (2026) https://BioRender.com/xducuw9 . **b**. Timeline for induction of *Trim47* deletion in EC following tamoxifen injection in adult mice (2 months). Behavioral tests, brain function assessment and qPCR screening were performed on adults *Trim47* $^{iEC-WT}$ and *Trim47* $^{iEC-KO}$ males. Created in in BioRender. Duplaa, C. (2026). https://BioRender.com/zo1kpb6. **c.** Body weight expressed in grams of 3 months males (n=8 *Trim47* $^{iEC-WT}$ and n=7 *Trim47* $^{iEC-KO}$). **c.** Body weight expressed in grams of 5 months males (n=8 *Trim47* $^{iEC-WT}$ and n=7 *Trim47* $^{iEC-KO}$). All graphical data are mean ± s.d.

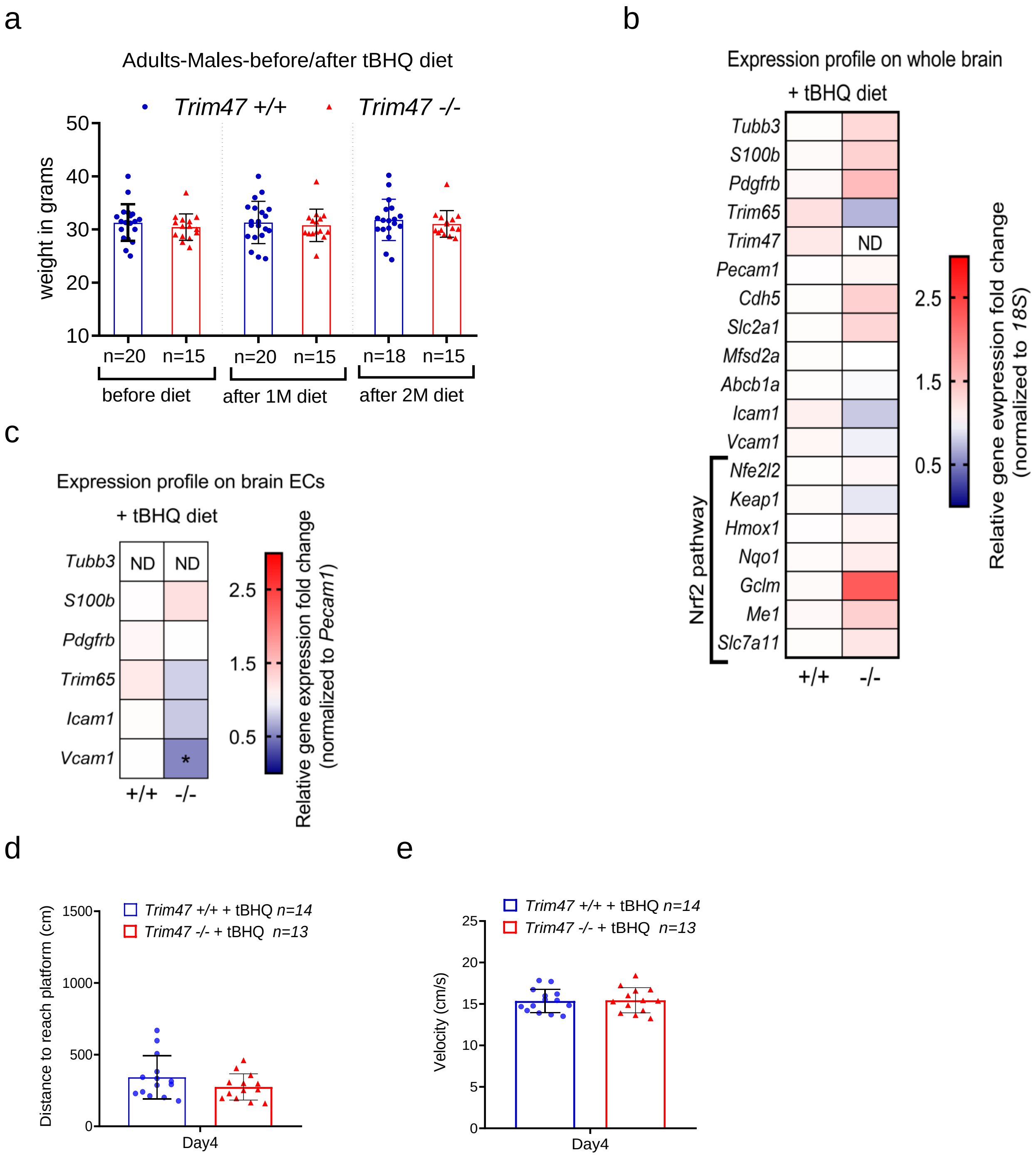

Supplementary Figure 14: Generation and characterization of mice deleted for *Trim47* specifically in endothelial cells. **a.** Monitoring of mouse body weight (in grams) before, after 1 month or 2 months of tBHQ diet showing no weight loss over time. **b-c**. qPCR screening on (**b**) whole brain and (**c**) brain EC isolated from *Trim47+/+ and Trim47-/-* males (8-10 months) with tBHQ diet (n=5 replicates/genotype). *: p<0.5; Mann-Whitney. ND: not detectable. **d-e.** Water maze test was performed on *Trim47+/+ and Trim47-/-* males with tBHQ diet (n=14 *Trim47+/+* + tBHQ and n=13 *Trim47+/+* + tBHQ). (**d**) Graph shows the distance to reach platform (expressed in cm) for the day 4. (**e**) Data represent the velocity expressed as cm per second (swimming speed) of each mouse at day 4 during water maze. All Graphical data are mean ± s.d.

	WMH					BG-PVS					WM-PVS				
	**beta**	**se**	**pval**	**pFDR**	**Qpval**	**beta**	**se**	**pval**	**pFDR**	**Qpval**	**beta**	**se**	**pval**	**pFDR**	**Qpval**
HMOX1	0,001	0,023	9,79E-01	1,00E+00	2,64E-01	0,044	0,019	1,84E-02	2,02E-01	1,58E-01	0,039	0,019	3,75E-02	3,72E-01	7,34E-01
GCLM	0,015	0,031	6,36E-01	1,00E+00	3,54E-01	0,048	0,028	8,33E-02	5,83E-01	4,99E-01	0,042	0,028	1,26E-01	6,30E-01	5,16E-01
TXNRD1	0,053	0,025	3,72E-02	3,72E-01	2,92E-01	0,053	0,02	6,41E-03	7,69E-02	4,91E-01	0,07	0,02	3,29E-04	4,94E-03	**3,06E-02**
GSR	0,042	0,061	4,88E-01	1,00E+00	3,62E-01	0,156	0,054	3,65E-03	**4,75E-02**	8,54E-01	0,063	0,053	2,34E-01	9,36E-01	9,36E-01
PRDX6	-0,036	0,018	4,76E-02	3,81E-01	4,64E-01	0,029	0,01	3,08E-03	**4,13E-02**	5,65E-01	0,017	0,01	8,94E-02	5,83E-01	5,75E-02

Supplementary Table 1: Association analysis of protein levels in plasma with MRI markers of cSVD, meta-analysis of 3C-Dijon and the UK Biobank (N=5,523). WMH: white matter hyperintensities, PVS: perivascular spaces; BG: basal ganglia, WM: white matter.

Target	Company	Sequence
siControl	Eurogentec	SR-CL000-005
siTRIM47 #1	Eurogentec	CCAGGGACUAUUUCCUCAA55
siTRIM47 #2	Eurogentec	GCAGCUGUUUGGAACCAAA55
siNRF2	Eurogentec	AUUGAUGUUUCUGAUCUAUCACU55

Supplementary Table 2: Sequences of siRNA used for HBMEC and HeLa transfection

Antibody (host)	Company	Catalogue number	Application and dilution
beta actin (rabbit)	Santa Cruz	sc-130656 (N21)	WB (HBMEC, 1/30000)
CD68 (rat)	Biolegend	137001	IF (mouse brain, 1/200)
Claudin5-488 (mouse)	ThermoFisher	352588	IF (mouse brain, 1/200)
ERG (rabbit)	Abcam	ab92513	IF (mouse retina, 1/200)
Fibrinogen (rabbit)	Dako	A0080	IF (mouse brain, 1/200)
GFAP (rabbit)	ThermoFisher	OPA1-06100	IF (mouse brain, 1/400)
Glut1 (rabbit)	ThermoFisher	PA1-1063	IF (mouse brain, 1/200)
HO1 (mouse)	Enzo life Science	ADI-OSA-110	WB (HBMEC, 1/1000)
HO1 (rabbit)	Proteintech	10701-1-AP	IF (mouse brain, 1/200)
IBA1 (rabbit)	Fujifilm Wako	019-19741	IF (mouse brain, 1/200)
Isolectin B4-FITC	Sigma	L 2895	IF (mouse retina, 1/200)
KEAP1 (rabbit)	Abcam	ab227828	WB (HBMEC, 1/1000), Co-IP (Hek293, 2 µg for 500 µg of protein lysates)
Myelin Basic Protein (rat)	Abcam	ab7349	IF (mouse brain, 1/200)
Myc (mouse)	Upstate	05-419	WB (Hek293, 1/1000)
Myc (mouse)	Millipore	05-724	Co-IP (Hek293, 2 µg for 500 µg of protein lysates)
NeuN (rabbit)	Millipore	ABN78	IF (mouse brain, 1/200)
NRF2 (rabbit)	Abcam	ab62352	WB (HBMEC, 1/1000)
Podocalyxin (goat)	R&D Systems	AF1556	IF (mouse brain, 1/400)
TRIM47 (rabbit)	Invitrogen	PA5-110521	WB (HBMEC , 1/1000)
TRIM47(rabbit)	Proteintech	26885-1-AP	WB (mouse brain, 1/1000)
α-tubulin (mouse)	Sigma	T5168	WB (HBMEC, 1/30000)
Ubiquitinylated proteins (mouse)	Sigma	04-263	Co-IP (Hek293, 2 µg for 500 µg of protein lysates)

Supplementary Table 3: List of antibodies used for this study on HBMEC and mouse tissue. IF: Immunofluorescence; WB: Western-Blot, Co-IP: co-immunoprecipitation. Dilution of the antibodies used for each specific application is specified in brackets.

Target	Forward	Reverse
PPIA/cyclophilin	AGCTAGACTTGAAGGGGAATG	ATTTCTTTTGACTTGCGGGC
TRIM47	TGAGCAGTCCAAAGTCCTGA	CTACGGCTGCACTCTTGATG
NFE2L2/NRF2	CACATCCAGTCAGAAACCAGTGG	GGAATGTCTGCGCCAAAGCTG
KEAP1	CCAACTTCGCTGAGCAGATT	GCTGATGAGGGTCACCAGTT
HMOX1/HO1	CCAGGCAGAGAATGCTGAGTTC	AAGACTGGGCTCTCCTTGTTGC
NQO1	GAAGAGCACTGATCGTACTGGC	GGATACTGAAAGTTCGCAGGG

Supplementary Table 4: List of human oligonucleotides used for qPCR

Target	Forward	Reverse
Tubb3/Tuj1	TCAGCGATGAGCACGGCATA	CACTCTTTCCGCACGACATC
S100b	CTGGAGAAGGCCATGGTTGC	CTCCAGGAAGTGAGAGAGCT
Gfap	AACCGCATCACCATTCCTGT	TGGCAGGGCTCCATTTTCAA
Cd11b/Itgam	TACTTCGGGCAGTCTCTGAGTG	ATGGTTGCCTCCAGTCTCAGCA
Pdgfrb	GCTAGCTGGTTGGCTAGCTG	CTTCCGGTGTCTAAATGTGGGT
Anpep	CGAACACCGTTTATCTGGACCTG	AGCCCATCTGTAGAATCCAGCG
Trim65	AGGAGCAACGCAGTCGGATTGA	GCCTGCTTCTTGGCTACCTCTA
Trim47	CTACAGAAACTCGGCTCAGAAGAT	GACTCCGGGTAGTTGATGGG
Pecam1	TCATTGGAGTGGTCATCGCC	TGTTGGAGTTCAGAAGTGGAGCAG
Cdh5	CCTGTAGGGAAAGAGTCCATTGTG	ACTTGACCGTGATGTTGGCG
Slc2a1/Glut1	TCTCTGTCGGCCTCTTTGTT	GCAGAAGGGCAACAGGATAC
Abcb1a	TCCTCACCAAGCGACTCCGATA	ACTTGAGCAGCATCGTTGGCGA
Mfsd2a	GCTCTGTCACCTCCTCACTG	ACGTTTCTACATTAGTGTTCCGAG
Nfe2l2/Nrf2	TAGATGACCATGAGTCGCTTGC	GCCAAACTTGCTCCATGTCC
Keap1	ATCCAGAGAGGAATGAGTGGCG	TCAACTGGTCCTGCCCATCGTA
Hmox1/Ho1	CACTCTGGAGATGACACCTGAG	GTGTTCCTCTGTCAGCATCACC
Nqo1	AGGATGGGAGGTACTCGAATC	AGGCGTCCTTCCTTATATGCTA
Gclm	AGGAGCTTCGGGACTGTATCC	GGGACATGGTGCATTCCAAAA
Me1	GTCGTGCATCTCTCACAGAAG	TGAGGGCAGTTGGTTTTATCTTT
Slc7a11	CTTTGTTGCCCTCTCCTGCTTC	CAGAGGAGTGTGCTTGTGGACA
Icam1	TGGCCTGGGGGATGCACACT	CCACCGGGCTGTAGGTGGGT
Vcam1	CGTACACCATCCGCCAGGCA	TAGAGTGCAAGGAGTTCGGGCG
Il1β	TGGACCTTCCAGGATGAGGACA	GTTCATCTCGGAGCCTGTAGTG
Il6	CACTTCACAAGTCGGAGGCT	CTGCAAGTGCATCATCGTTGT
Tnfα	CCTCCTGGCCAACGGCATGG	GCAGGGGCTCTTGACGGCAG
Ifnar1	CGCGGAGGGCCTAGC	GTGGCTGCTCCACTTTAGGG
Ifnar2	GAGCCTAGAGACTATCACACCG	GCCCTCCAACCACTTATCTG
Isg15	TGACTGTGAGAGCAAGCAGC	CCCCAGCATCTTCACCTTTA
Cldn5	ACGGGAGGAGCGCTTTAC	GTTGGCGAACCAGCAGAG
Cldn11	GCCTGGAGTGGCCAAGTA	AGATGGTGGCGACAATGG
Ocln	GTCCGTGAGGCCTTTTGA	GGTGCATAATGATTGGGTTTG
Tjp1	AAGTTGGCAAGAGAGGAGCC	CAACCGCATTTGGCGTTACA
Plvap	GTTGACTACGCGACGTGAGATG	AGCTGTTCCTGGCACTGCTTCT
18S	CGCGGTTCTATTTTGTTGGT	AGTCGGCATCGTTTATGGTC

Supplementary Table 5: List of mouse oligonucleotides used for qPCR

Supplementary Figure 15: Uncropped immunoblots underlying Figures

Uncropped immunoblots underlying Figure 1d

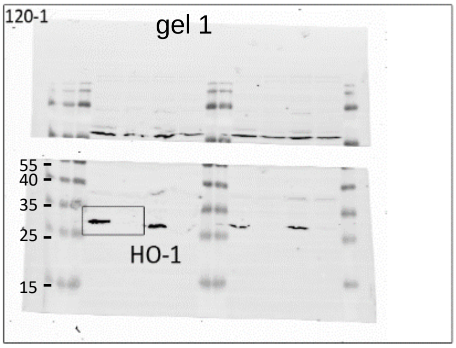

12% Acrylamide Gel

Channel 700nm Low Exposure

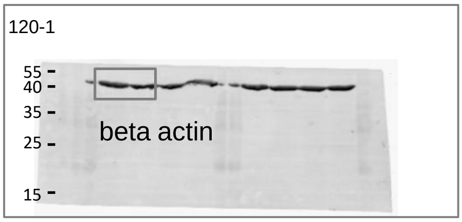

12% Acrylamide Gel

Channel 800nm

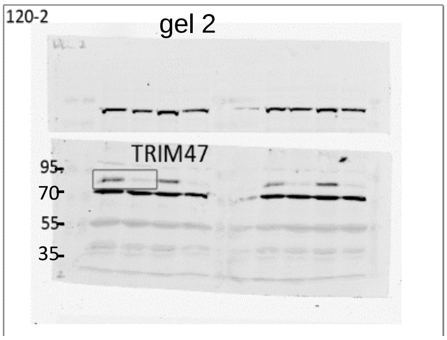

6% Acrylamide Gel

Channel 800nm Low Exposure

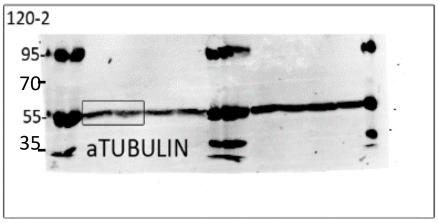

6% Acrylamide Gel

Channel 700nm

Uncropped immunoblots underlying Figure 2d

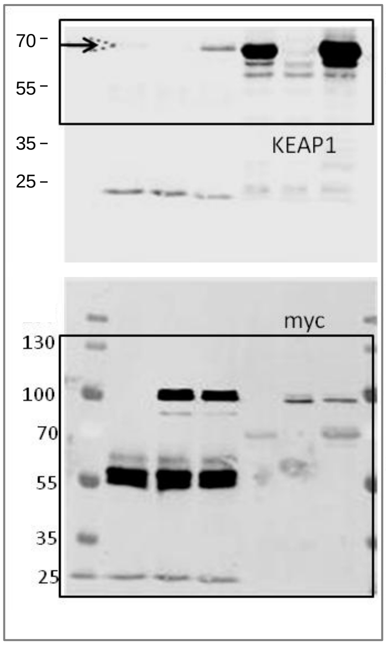

Channel 800nm

Channel 700nm

Uncropped immunoblots underlying Supplementary Figure 3a

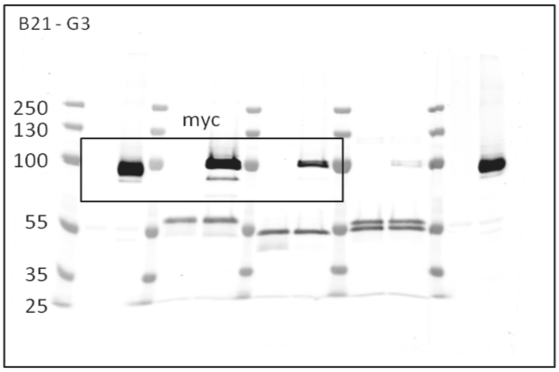

Channel 700nm

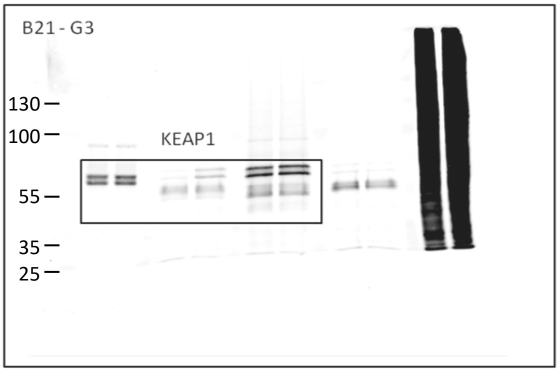

Channel 800nm

Supplementary Figure 16: Uncropped immunoblots underlying Figures

Uncropped immunoblots underlying Supplementary Figure 3b

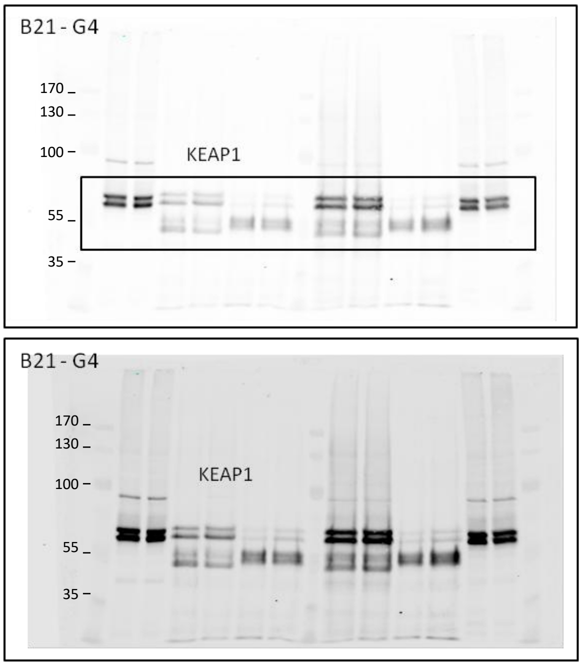

Channel 800nm

Channel 800nm High exposure

Uncropped immunoblots underlying Figure 2e

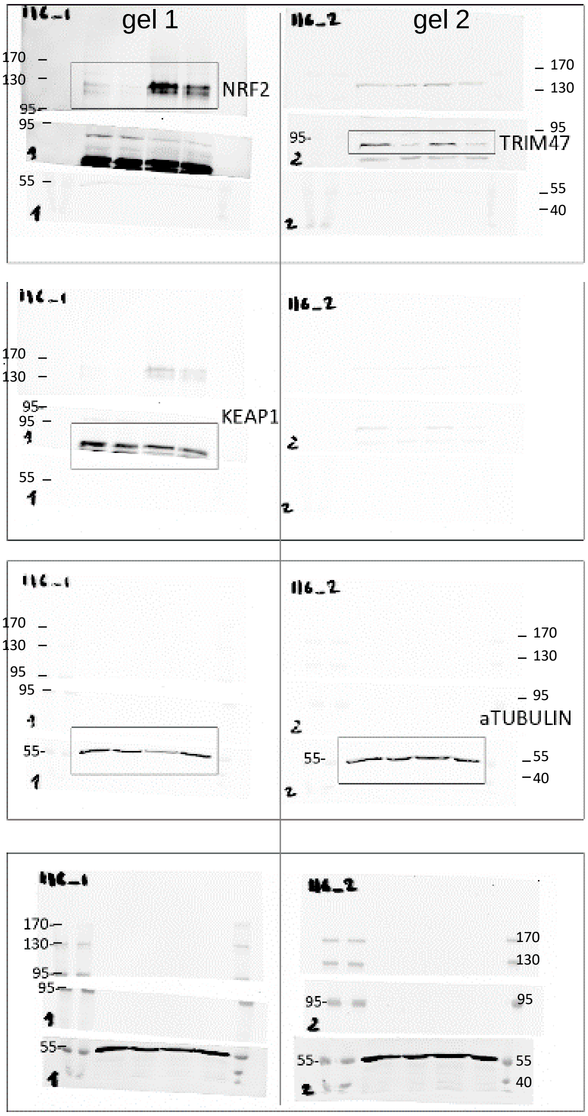

6% Acrylamide
Channel 800nm

6% Acrylamide
Channel 800nm Low Exposure

6% Acrylamide
Channel 700nm Low Exposure

6% Acrylamide
Channel 700nm
