# Supplementary material for: Endothelial TRIM47 regulates blood-brain barrier integrity and cognition via the KEAP1/NRF2 signalling pathway in mice
Source: Commun Biol. 2026 Feb 10;9:399. doi: 10.1038/s42003-026-09628-5 (PMC13000003; doi:10.1038/s42003-026-09628-5)
Supplement: Supplementary file 3 — Description of Additional Supplementary File [file 42003_2026_9628_MOESM3_ESM.pdf]

Description of Additional Supplementary Files

File name: Supplementary Data 1

Description: source data file
