# Supplementary material for: Endothelial TRIM47 regulates blood-brain barrier integrity and cognition via the KEAP1/NRF2 signalling pathway in mice
Source: Commun Biol. 2026 Feb 10;9:399. doi: 10.1038/s42003-026-09628-5 (PMC13000003; doi:10.1038/s42003-026-09628-5)
Supplement: Supplementary file 5 — Reporting Summary [file 42003_2026_9628_MOESM5_ESM.pdf]

nature portfolio

Corresponding author(s): Dr Claire PEGHAIRE, COMMSBIO-25-4660-B

Last updated by author(s): Jan 12, 2026

Reporting Summary

Nature Portfolio wishes to improve the reproducibility of the work that we publish. This form provides structure for consistency and transparency in reporting. For further information on Nature Portfolio policies, see our Editorial Policies and the Editorial Policy Checklist.

Statistics

For all statistical analyses, confirm that the following items are present in the figure legend, table legend, main text, or Methods section.

n/a	Confirmed	
☐	☒	The exact sample size (*n*) for each experimental group/condition, given as a discrete number and unit of measurement
☐	☒	A statement on whether measurements were taken from distinct samples or whether the same sample was measured repeatedly
☐	☒	The statistical test(s) used AND whether they are one- or two-sided<br>*Only common tests should be described solely by name; describe more complex techniques in the Methods section.*
☒	☐	A description of all covariates tested
☐	☒	A description of any assumptions or corrections, such as tests of normality and adjustment for multiple comparisons
☐	☒	A full description of the statistical parameters including central tendency (e.g. means) or other basic estimates (e.g. regression coefficient) AND variation (e.g. standard deviation) or associated estimates of uncertainty (e.g. confidence intervals)
☒	☐	For null hypothesis testing, the test statistic (e.g. *F*, *t*, *r*) with confidence intervals, effect sizes, degrees of freedom and *P* value noted<br>*Give P values as exact values whenever suitable.*
☒	☐	For Bayesian analysis, information on the choice of priors and Markov chain Monte Carlo settings
☒	☐	For hierarchical and complex designs, identification of the appropriate level for tests and full reporting of outcomes
☒	☐	Estimates of effect sizes (e.g. Cohen's *d*, Pearson's *r*), indicating how they were calculated

Our web collection on statistics for biologists contains articles on many of the points above.

Software and code

Policy information about availability of computer code

Data collection	Odyssey imaging system (Li-COR, ScienceTec) and Odyssey ver.3 software for immunoblots; Zen software (Zeiss) for widefield and confocal microscopy; Spark® Multimode Microplate Reader and software (Tecan) for luciferase assay and BBB pemeability, QuantStudio™ 3 qPCR System and software (Thermo Fisher Scientific) for qPCR, EthoVisionXT 16 software (Noldus) for behavioural testing.
Data analysis	Zen blue (version 3.5, Zeiss) and ImageJ (version 2.3.0, NIH) for image analysis; Prism ver. 8.0 (GraphPad) for statistical analysis. OlinkAnalyze R package (v4.3.1, https://github.com/Olink-Proteomics/OlinkRPackage), ggplot2 R package (v3.5.2, https://github.com/tidyverse/ggplot2), forestplot R package (v3.1.7, https://cran.r-project.org/web/packages/forestplot) and SHIVA-PVS algorithm (https://github.com/pboutinaud/SHIVA_PVS, T1.PVS/v1) were used for Olink proteomic human data analysis.

For manuscripts utilizing custom algorithms or software that are central to the research but not yet described in published literature, software must be made available to editors and reviewers. We strongly encourage code deposition in a community repository (e.g. GitHub). See the Nature Portfolio guidelines for submitting code & software for further information.

Data

Policy information about availability of data

All manuscripts must include a data availability statement. This statement should provide the following information, where applicable:

- Accession codes, unique identifiers, or web links for publicly available datasets
- A description of any restrictions on data availability
- For clinical datasets or third party data, please ensure that the statement adheres to our policy

The raw reads for bulk RNA-sequencing in FASTQ format have been deposited in Gene Expression Omnibus (GEO) database under accession number: GSE279052. The UK Biobank data used in this study were obtained from the UK Biobank under applications no. 94113. Proteomic and MRI data are available upon reasonable request through the UK Biobank access procedures (www.ukbiobank.ac.uk). Due to confidentiality and ethical restrictions, individual-level proteomic data from the 3C cohort are not publicly available but can be accessed upon reasonable request to the 3C steering committee. Contact details for the data access committee are available via the study coordinating center.
All other data supporting the findings of this work are available within the paper and its Supplementary Information file including Source data file. Uncropped immunoblots are presented in Supplementary Figures 15 and 16. Any other data are available from the corresponding author upon reasonable request.

Research involving human participants, their data, or biological material

Policy information about studies with human participants or human data. See also policy information about sex, gender (identity/presentation), and sexual orientation and race, ethnicity and racism.

Reporting on sex and gender	NA
Reporting on race, ethnicity, or other socially relevant groupings	NA
Population characteristics	NA
Recruitment	NA
Ethics oversight	Informed consent of participants was obtained. All ethical regulations relevant to human research participants were followed. Study protocols were approved by the appropriate boards at their respective institutions: ethics committee of the University Hospital of Kremlin-Bicêtre for 3C-Dijon, and the National Research Ethics Service Committee North West-Haydock (reference 11/NW/0382) for UK Biobank.

Note that full information on the approval of the study protocol must also be provided in the manuscript.

Field-specific reporting

Please select the one below that is the best fit for your research. If you are not sure, read the appropriate sections before making your selection.

☒ Life sciences ☐ Behavioural & social sciences ☐ Ecological, evolutionary & environmental sciences

For a reference copy of the document with all sections, see nature.com/documents/nr-reporting-summary-flat.pdf

Life sciences study design

All studies must disclose on these points even when the disclosure is negative.

Sample size	In vitro experiments were performed at least three independent times. Sample size in all experiments was estimated based to previous published experiments. At least three or more mice were used in animal experiment to obtain statistical analysis and to comply with the Animal research 3Rs (replacement, reduction, refinement) principle. In this study, the statistic analysis was obtained using two-sided t-test , Mann-whitney, One-Way or Two-WAY ANOVA and the values represents means plus minus standard error of the mean.
Data exclusions	Animals with a glaucoma or that were not able to find and /or see the visible platform during the 2 blocks of initial visualisation phase of the Water Maze experiments were excluded from the behavioural mouse cohorts.
Replication	Experiments were performed at least three independent times, all experimental findings were reproducible.
Randomization	Mice were randomly allocated to experimental groups according to their genotype using the Excel function ALEA(). Males and females were analyzed separately for the behavioral assessment. Randomly selected samples were allocated to in vitro experimental groups.
Blinding	Blinding was used whenever possible. However, since the in vitro experiments were performed and analyzed by the same person, blinding was not feasible for these. For the in vivo experiments, the person performing the behavioral tests was blinded to the genotype and treatment of the mice.

Reporting for specific materials, systems and methods

We require information from authors about some types of materials, experimental systems and methods used in many studies. Here, indicate whether each material, system or method listed is relevant to your study. If you are not sure if a list item applies to your research, read the appropriate section before selecting a response.

Materials & experimental systems

n/a	Involved in the study
☐	☒ Antibodies
☐	☒ Eukaryotic cell lines
☒	☐ Palaeontology and archaeology
☐	☒ Animals and other organisms
☒	☐ Clinical data
☒	☐ Dual use research of concern
☒	☐ Plants

Methods

n/a	Involved in the study
☒	☐ ChIP-seq
☒	☐ Flow cytometry
☒	☐ MRI-based neuroimaging

Antibodies

Antibodies used	CD68 (rat), Biolegend, 137001; Claudin5-488 (mouse), ThermoFisher, 352588; Fibrinogen (rabbit), Dako, A0080; GFAP (rabbit), ThermoFisher, OPA1-06100; Glut1 (rabbit), ThermoFisher, PA1-1063; HO1 (mouse), Enzo life Science, Enzo life Science; IBA1 (rabbit), Fujifilm Wako, 19-19741; Isolectin B4-FITC, Sigma, L 2895; KEAP1 (rabbit), Abcam, ab227828; Myelin Basic Protein (rat), Abcam, ab7349 ; Myc (mouse), Millipore, 05-724 ; NeuN (rabbit), Millipore, ABN78 ; NRF2 (rabbit), Abcam, ab62352 ; Podocalyxin (goat), R&D Systems, AF1556 ; TRIM47 (rabbit), Invitrogen, PA5-110521 ; TRIM47(rabbit), Proteintech, 26885-1-AP ; α-tubulin (mouse), Sigma, T5168: beta actin, Santa cruz, sc-130656 (N21).
Validation	Specificity of TRIM47, NRF2 and KEAP1 antibodies was validated in vitro in endothelial cells treated with TRIM47, NRF2 or KEAP1 siRNA. Every antibodies used in this study was validated by their suppliers for the specific application.

Eukaryotic cell lines

Policy information about cell lines and Sex and Gender in Research

Cell line source(s)	HBMEC from SicenCell (#1000) and cultured in Endothelial Cell Growth Medium-2 media (EGM-2) (Lonza). These primary cells were used up to passage 6 maximum. HeLa (ATCC CCL-2) and Hek293 (ATCC CRL-1573) were cultured in DMEM (Dulbecco's Modified Eagle Medium, Thermo Fisher Scientific) supplemented with 10% FBS (fetal bovine serum) and 1% penicillin-streptomycin (Thermo Fisher Scientific).
Authentication	No further authentication has been performed. A certificate of analysis and purity is provided for each cell lot purchased from the companies. No further authentication has been performed. A certificate of analysis and purity is provided for each cell lot purchased from the companies. HBMEC from ScienCell Research Laboratories are isolated from human brain. HBMEC are cryopreserved at passage one and delivered frozen. Each vial contains >5 x 105 cells in 1 ml volume. HBMEC are characterized by immunofluorescence with antibodies specific to vWF and/or CD31 (PECAM1).
Mycoplasma contamination	Tested negative for HIV-1, HBV, HCV, mycoplasma, bacteria, yeast, and fungi by the company. Tested for mycoplasma by qPCR once a month.
Commonly misidentified lines (See ICLAC register)	The study did not use misidentified lines.

Animals and other research organisms

Policy information about studies involving animals; ARRIVE guidelines recommended for reporting animal research, and Sex and Gender in Research

Laboratory animals	Trim47 full KO mice (C57BL/6N-Trim47em1(IMPC)Bay/Mmmh) were purchased from the national public repository system for mutant mice Mutant Mouse Resource and Research Center. The inducible endothelial-specific Trim47 knockout mouse model (Trim47iEC-KO) was generated by breeding Trim47fl/fl mice (strain ID: T005408, GemPharmtech) with Cdh5(PAC)-CreERT2 mice (69). Endothelial deletion of Trim47 was induced in adult mice 2 months old), by tamoxifen injection (5648, Sigma) (five injections of 0.5 mg daily). All experiments were conducted using littermate controls denoted in the text as Trim47+/+ for the full KO mice and as Trim47iEC-WT for the endothelial-specific deleted mice line. Animlas from 1 week and up to 12 months (male and female) mice were used for this study.
Wild animals	The study did not involved wild animals.
Reporting on sex	Both males and females wer used for this study since cebr

Field-collected samples	The study did not involved samples collection from the field.
Ethics oversight	Animal experiments were approved by the local Animal Care and Use Committee of the Bordeaux University CEEA50 (IACUC protocol #36971). We have complied with all relevant ethical regulations for animal use. Mouse studies were conducted in accordance with the guidelines from Directive 2010/63/EU of the European Parliament on the protection of animals used for testing and research. General procedures were conducted in mice according to ARRIVE guidelines (https://www.nc3rs.org.uk/arrive-guidelines). All animals used were retained on a C57BL/6 background and both male and female mice were used for experiments. Animals received pre-emptive analgesia with buprenorphine (Vetergesic®) to minimize pain and distress. Mice were euthanized by administration of a lethal dose of injectable anesthetic (ketamine/xylazine), performed by authorized and trained personnel. For blood–brain barrier assessment procedures, an anesthetic eye drop (tetracaine 1%, single dose) was applied to the eye 5 minutes prior to injection, and fluorescent tracers were administered intravenously via retro-orbital injection under inhalation anesthesia (2% isoflurane). For brain histology procedures, animals were placed under deep general anesthesia induced by ketamine/xylazine and euthanized by transcardial perfusion with PBS followed by PFA, resulting in exsanguination and confirmed death. For brain endothelial cell isolation, mice were euthanized by cervical dislocation performed under deep general anesthesia induced by ketamine/xylazine.

Note that full information on the approval of the study protocol must also be provided in the manuscript.

Plants

Seed stocks	NA
Novel plant genotypes	NA
Authentication	NA
